# Quercetin in Idiopathic Pulmonary Fibrosis and Its Comorbidities: Gene Regulatory Mechanisms and Therapeutic Implications

**DOI:** 10.3390/genes16080856

**Published:** 2025-07-23

**Authors:** Verónica Rocío Vásquez-Garzón, Juan Manuel Velázquez-Enríquez, Jovito Cesar Santos-Álvarez, Alma Aurora Ramírez-Hernández, Jaime Arellanes-Robledo, Cristian Jiménez-Martínez, Rafael Baltiérrez-Hoyos

**Affiliations:** 1 Laboratorio de Fibrosis y Cáncer, Facultad de Medicina y Cirugía, Universidad Autónoma Benito Juárez de Oaxaca, Ex Hacienda de Aguilera S/N, Sur, San Felipe del Agua, Oaxaca C.P. 68020, Mexico; vrvasquezga@secihti.mx (V.R.V.-G.); juanmanuelvela_enriquez@cecad-uabjo.mx (J.M.V.-E.); jovitocesarsa@cecad-uabjo.mx (J.C.S.-Á.); aramih_09@cecad-uabjo.mx (A.A.R.-H.); 2 Directorate of Support for the Consolidation of the Scientific and Humanistic Community, Secretariat of Science, Humanities, Technology and Innovation—SECIHTI, Mexico City 03940, Mexico; jarellanes@inmegen.gob.mx; 3 SECIHTI—Facultad de Medicina y Cirugía, Universidad Autónoma Benito Juárez de Oaxaca, Ex Hacienda de Aguilera S/N, Sur, San Felipe del Agua, Oaxaca C.P. 68020, Mexico; 4 Centro de Investigación en Nutrición y Alimentación, Licenciatura en Nutrición, Universidad del Istmo, Carretera Transísmica Juchitán, la ventosa km. 14, La Ventosa, Oaxaca 70102, Mexico; 5 Laboratory of Liver Diseases, National Institute of Genomic Medicine—INMEGEN, Mexico City 14610, Mexico; 6 Departamento de Ingeniería Bioquímica, Escuela Nacional de Ciencias Biológicas, Instituto Politécnico Ncional, Unidad Profesional Adolfo López Mateos, Zacatenco, Av. Wilfrido Massieu Esq. Cda. Miguel Stampa S/N, Alcaldía Gustavo A. Madero, Mexico City 07738, Mexico; cjimenezh@ipn.mx

**Keywords:** epigenetic modulation, microRNAs, therapeutic potential, oxidative stress, inflammatory signaling pathways

## Abstract

Idiopathic pulmonary fibrosis (IPF) is a chronic and progressive interstitial lung disease associated with high morbidity and mortality. Both pulmonary and extrapulmonary comorbidities significantly influence disease progression and patient outcomes. Despite current therapeutic options, effective treatments remain limited. Quercetin, a naturally occurring flavonoid, has emerged as a promising compound due to its antioxidant, anti-inflammatory, and antifibrotic properties. Preclinical and clinical studies have demonstrated its ability to modulate key molecular pathways involved in IPF, including Nrf2, SIRT1/AMPK, and the regulation of fibrosis-associated microRNAs (miRNAs). Furthermore, quercetin shows therapeutic potential across a range of IPF-related comorbidities, including chronic obstructive pulmonary disease, pulmonary hypertension, lung cancer, cardiovascular disease, diabetes, and psychiatric disorders. Under these conditions, quercetin acts via epigenetic modulation of miRNAs and regulation of oxidative stress and inflammatory signaling pathways. This review highlights the multifunctional role of quercetin in IPF and its comorbidities, emphasizing its gene regulatory mechanisms and potential as an adjunctive or alternative therapeutic strategy.

## 1. Introduction

Idiopathic pulmonary fibrosis (IPF) is a chronic, progressive lung disease characterized by excessive formation of scar tissue in the lungs, leading to an irreversible decline in lung function and ultimately to respiratory failure and death [1,2]. IPF represents a significant clinical challenge, with a median survival of 3–5 years approximately after diagnosis [3]. Despite advances in understanding and managing IPF, treatment options remain limited.

Comorbidity in IPF refers to the presence of one or more disorders or diseases in addition to IPF, which may interact with or exacerbate it. Several studies have shown that patients with IPF have a higher incidence and prevalence of both pulmonary and extrapulmonary comorbidities [4]. These comorbidities encompass a wide range of conditions that significantly impact the clinical course of the disease and the patient’s quality of life. Among the most frequent comorbidities in patients with IPF are chronic obstructive pulmonary disease (COPD), pulmonary hypertension (PH), and lung cancer (LC). These pulmonary conditions not only complicate the management of IPF but also contribute to an increased symptomatic burden and rapid deterioration of lung function. In addition, the presence of gastroesophageal reflux may exacerbate respiratory symptoms and contribute to the progression of fibrosis [5,6,7,8,9].

Extrapulmonary comorbidities also play crucial roles in IPF. Cardiovascular diseases (CVDs), such as coronary artery disease (CAD), are common in these patients and increase the risk of adverse cardiovascular events. Diabetes adds a layer of complexity to treatment, while acute exacerbations can precipitate rapid clinical deterioration. In addition, psychiatric diseases such as anxiety, depression, and gastrointestinal issues such as dyspepsia and irritable bowel syndrome, are also prevalent and adversely affect patients’ quality of life [5,6,7,8,9].

The influence of these comorbidities on IPF cannot be underestimated, as they contribute significantly to increased morbidity and mortality rates. Therefore, early identification and appropriate treatment of these comorbidities and associated complications are essential. Thus, addressing these conditions can significantly improve overall outcomes, including quality of life and patient survival, which is crucial for optimizing the care of patients with IPF.

## 2. Treatments for IPF

The therapeutic approaches for IPF are focused on pharmacological and nonpharmacological strategies. In the pharmacological field, two drugs are currently approved by the Food and Drug Administration (FDA), namely pirfenidone and nintedanib. Pirfenidone exerts antifibrotic effects by inhibiting collagen synthesis, fibroblast proliferation, and the expression of profibrotic cytokines such as transforming growth factor beta (TGF-β) and tumor necrosis factor-alpha (TNF-α); meanwhile nintedanib, on the other hand, acts as a multityrosine kinase inhibitor, blocking signaling from receptors associated with key growth factors involved in the progression of fibrosis, such as platelet-derived growth factor (PDGF), fibroblast growth factor (FGF), and vascular endothelial growth factor (VEGF). Despite these therapeutic advances, lung transplantation remains the only intervention capable of substantially modifying the prognosis of patients with IPF [10].

However, both pirfenidone and nintedanib have been shown to slow the decline in lung function, and they neither halt disease progression nor significantly improve survival [11]. Furthermore, their adverse effects—which can range from mild symptoms such as nausea and fatigue to more serious complications such as severe diarrhea and hepatotoxicity—represent a significant clinical challenge, as they can compromise therapeutic adherence and negatively impact patients’ quality of life [12].

Regarding nonpharmacological strategies, supplemental oxygen therapy is recommended, especially in patients who present with exercise-induced oxygen desaturation, even when resting oxygen levels remain within normal ranges. Pulmonary rehabilitation, which includes aerobic training, strength and flexibility exercises, palliative care, and psychological support, is also recommended [13]. Furthermore, several studies have reported a significant reduction in muscle mass and body weight in patients with IPF, which is associated with reduced functional capacity and low levels of physical activity. In this context, specialized nutritional intervention represents an essential component of a multidisciplinary approach to improve the functional status and quality of life of these patients [14].

Therefore, despite advances in treating IPF, there is an urgent need to identify new therapies that improve the clinical outcomes and quality of life of affected patients.

## 3. Alternative Treatment for IPF and Its Comorbidities

**Figure 1:**
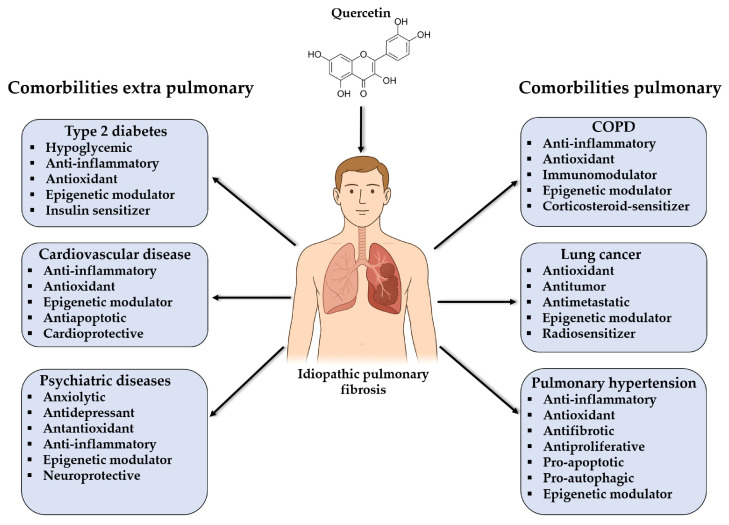
Illustration of pleiotropic therapeutic effects of quercetin on pulmonary and extrapulmonary comorbidities associated with IPF.

In IPF, epigenetic mechanisms have emerged as critical modulators of aberrant fibroblast activation and lung parenchyma remodeling. Particularly, microRNAs (miRNAs), which are small RNA sequences of 19–22 nucleotides capable of regulating gene expression through their complementarity with target sequences in mRNAs, stand out. It is estimated that miRNAs can modulate the expression of more than 50% of protein-coding genes, affecting key processes such as apoptosis, proliferation, and cell differentiation [15]. miRNAs such as miR-21, miR-29, and miR-200 are associated with profibrotic pathways including the TGF-β/Smad and Wnt/β-catenin pathways. For example, miR-21 is frequently overexpressed in fibrotic lung tissue, promoting the activation of myofibroblasts and the accumulation of extracellular matrix (ECM) [16]; on the other hand, the loss of miR-29 promotes the expression of collagen genes [17,18,19,20]. In this context, quercetin has been shown to modulate the expression of various miRNAs in preclinical models. Quercetin is a flavonoid with antioxidant, anti-inflammatory, antifibrotic, hypoglycemic, antiapoptotic, anxiolytic, antidepressant, neuroprotective, antiproliferative, pro-autophagy, antimetastatic, immunomodulatory, and epigenetic modulator properties, suggesting that part of its therapeutic effect could be mediated by the restoration of epigenetic balance in various comorbidities associated with IPF (Figure 1), which will be analyzed in this review.

## 4. Quercetin: Chemical Properties, Pharmacokinetics, and Mechanistic Insights

**Figure 2:**
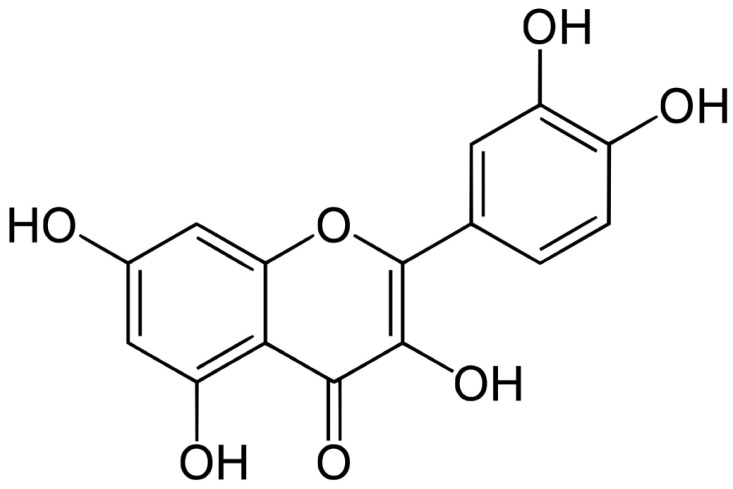
The chemical structure of quercetin.

Quercetin (3,3′,4′,5,7-pentahydroxyflavone) is a naturally occurring polyphenolic compound belonging to the flavonol subclass of flavonoids, with a molecular weight of 302.24 g/mol and a topological polar surface area of 127 Å^2^. Structurally, it consists of a central heterocyclic pyrone ring linked to two benzene rings, forming a classic flavone backbone (Figure 2) [21]. It appears as a yellow crystalline solid, insoluble in water but soluble in glacial acetic acid and alkaline aqueous solutions [22]. In plants, quercetin is commonly found as glycosylated derivatives (e.g., quercetin-3-glucoside), particularly in onions, apples, berries, tea, and capers. Once ingested, these glycosides are hydrolyzed and subsequently conjugated (via methylation, glucuronidation, sulfonation) in enterocytes and hepatocytes, producing metabolites such as quercetin-3-glucuronide and quercetin-3′-sulfate, which are the predominant forms found in plasma [23]. Despite its extensive biological potential—encompassing antioxidant, anti-inflammatory, antifibrotic, and anticancer activities—quercetin’s low aqueous solubility and extensive first-pass metabolism limit its bioavailability. While conjugated metabolites can reach plasma concentrations of 3.5–5.0 μmol/L, the absorption of unconjugated quercetin is inefficient, with peak levels under 0.33 μmol/L [24].

Mechanistically, quercetin exerts its effects by modulating multiple signaling pathways that are central to redox balance, inflammation, and fibrogenesis—most notably, Nrf2, AMPK, NF-κB, and SIRT1. These do not function in isolation but are interlinked in a hierarchical regulatory network, where SIRT1 activates AMPK, which in turn promotes Nrf2-driven transcription of antioxidant genes, while collectively suppressing NF-κB–mediated inflammatory responses [22,25,26]. This coordinated action underlies quercetin’s pleiotropic and tissue-protective effects. In addition to these canonical pathways, quercetin also modulates epigenetic regulators such as miRNAs. It has been shown to upregulate miR-16, miR-138-5p, and the let-7 family, and downregulate miR-21, miR-155, and miR-29, all of which are implicated in fibrosis, senescence, and chronic inflammation.

Although the systemic administration of quercetin results in widespread exposure, its ability to modulate miRNA expression across multiple organs likely reflects a combination of direct and indirect mechanisms. Tissue-specific effects may arise from differential expression of upstream signaling pathways (e.g., Nrf2, NF-κB, AMPK) and the local redox/inflammatory environment, which shape the cellular responsiveness to quercetin [27]. Furthermore, emerging evidence suggests that quercetin may influence the release and miRNA cargo of extracellular vesicles (EVs), allowing for inter-organ communication of regulatory signals through vesicle-mediated transport [28]. These aspects warrant further investigation through pharmacokinetic and tissue-targeted delivery studies, especially to optimize therapeutic precision and minimize off-target effects.

However, while the functional outcomes of miRNA modulation by quercetin have been consistently observed across multiple preclinical models, the precise molecular mechanisms remain poorly understood. To date, no direct evidence exists of quercetin binding to miRNA promoters, inducing chromatin remodeling, or modulating key miRNA-processing enzymes such as Dicer or Drosha. In contrast, other polyphenols like resveratrol and epigallocatechin gallate (EGCG) have been shown to directly bind to miRNAs (e.g., miR-33a and miR-122), providing a plausible model of direct interaction. Similar promoter or transcriptional activity assays for quercetin are lacking, highlighting a critical gap in mechanistic understanding [26,29]. Future studies should aim to explore whether quercetin affects miRNA expression via upstream signaling cascades, epigenetic enzyme modulation, or direct nucleic acid interaction.

Importantly, quercetin has been shown to exhibit a biphasic dose–response pattern (hormesis) in various models, including lung fibroblasts and metabolic tissues [30,31]. At low to moderate concentrations, it activates cytoprotective pathways such as Nrf2/HO-1 and AMPK, promoting antioxidant defenses and reducing inflammation. However, at higher doses, it may paradoxically induce oxidative stress, mitochondrial dysfunction, and apoptosis, particularly in sensitive cell types [32]. In animal models of IPF, antifibrotic activity has been observed at doses ranging from 25 to 100 mg/kg/day, whereas in humans, clinical studies suggest good tolerability up to 1000 mg/day, with occasional mild gastrointestinal effects [30,33]. These findings underscore the importance of dose optimization and formulation strategies to maximize therapeutic benefit while minimizing potential toxicity.

## 5. Quercetin and IPF

**Figure 3:**
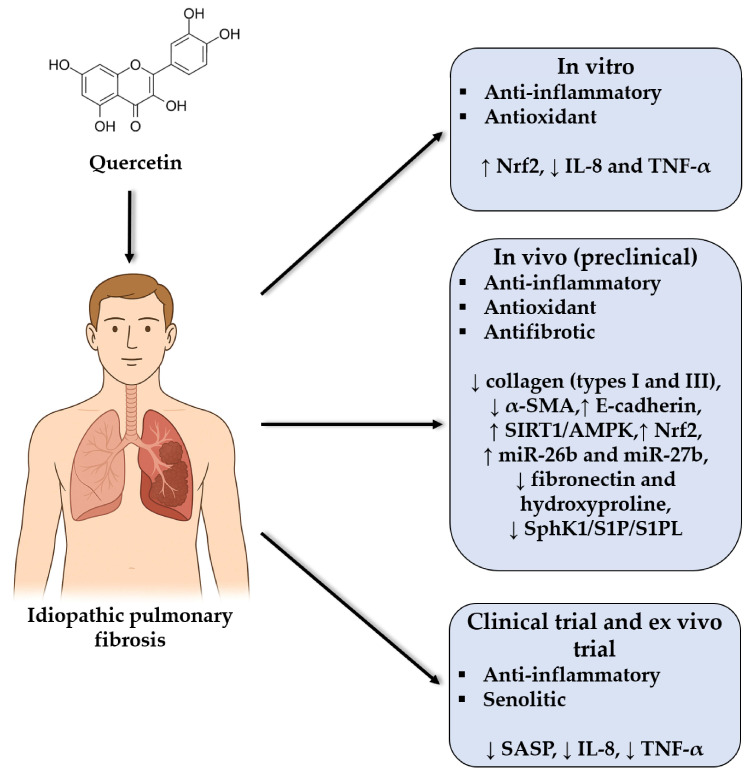
Schematic representation of the main therapeutic effects, signaling pathways and target molecules modulated by quercetin in in vitro, in vivo, ex vivo models and clinical trials in the context of idiopathic pulmonary fibrosis (IPF). The symbol “↓” indicates downregulation, and “↑” indicates upregulation.

Quercetin, a dietary flavonoid with well-documented antioxidant, anti-inflammatory, and senolytic properties, has shown promising effects in preclinical models and early phase clinical studies of IPF (Figure 3) [34,35,36]. In pilot clinical studies, the senolytic combination of dasatinib and quercetin has been demonstrated to be safe and feasible in patients with IPF, a disease closely associated with cellular aging. In a controlled clinical trial, 12 patients completed treatment without experiencing any serious adverse events; however, mild side effects, such as sleep disturbances and malaise, were reported, suggesting good tolerability. In another open-label study, which included 14 patients with IPF, significant improvement in physical function was observed, although no relevant changes in lung function were detected. Although the results for senescence biomarkers, such as senescence-associated secretory phenotypes (SASPs) are still preliminary, correlations with inflammatory mediators and ECM remodeling factors have been identified, reinforcing the need for larger clinical trials to validate the findings [34,37].

IPF is characterized by redox imbalance and a chronic inflammatory state. In this context, IPF patients have decreased total antioxidant capacity, as well as reduced levels of glutathione and uric acid. Ex vivo incubation of blood samples with quercetin significantly reduced the production of IL-8 and TNF-α, especially in samples from IPF patients. Additionally, in an in vitro model using BEAS-2B cells, quercetin activated the Nrf2 pathway and reduced the expression of proinflammatory cytokines in a concentration-dependent manner [35].

Several studies have evaluated the therapeutic potential of quercetin in murine models of bleomycin (BLM)-induced IPF, demonstrating that the antifibrotic effects of quercetin are mediated by different molecular mechanisms. In a study, quercetin decreased the expression of markers such as α-SMA and collagens I and III and increased the expression of E-cadherin. These effects are mediated by the activation of the SIRT1/AMP-activated protein kinase (AMPK) pathway and the induction of autophagy. These effects were reversed by the inhibitor EX-527, confirming the role of this pathway in the epithelial-to-mesenchymal transition [36]. In another study, quercetin was shown to reduce the levels of hydroxyproline, fibronectin, and S1P/SphK1/S1PL signaling in both lung tissue and TGF-β-stimulated fibroblasts. The overexpression of SphK1 abolished the antifibrotic effects of quercetin, indicating that its therapeutic action depends on the inhibition of the SphK1/S1P pathway [38]. Furthermore, in a study focusing on the Nrf2 pathway, mice fed a quercetin-supplemented diet presented increased expression of antioxidant genes and reduced lung inflammation and fibrosis. These effects were not observed in Nrf2-deficient mice, suggesting that this redox pathway is essential for quercetin activity [39].

In parallel, other studies performed in preclinical models of bleomycin-induced IPF have confirmed the antifibrotic effects of quercetin. In an experimental rat model, its impact on lung architecture and the expression of fibrosis-associated miRNAs, such as miR-26b and miR-27b, were evaluated. The results indicated that quercetin improves alveolar structure, decreases collagen accumulation, reduces α-SMA expression, and increases E-cadherin expression, in addition to restoring the levels of the aforementioned miRNAs [40].

Together, these findings support the therapeutic potential of quercetin as a multifunctional antifibrotic agent in IPF that is capable of reversing histological and molecular alterations, restoring redox balance, and attenuating the inflammatory process.

### 5.1. Pulmonary Comorbidities

#### 5.1.1. Quercetin and COPD

COPDs are a group of lung conditions that worsen over time and include emphysema, chronic bronchitis, minor airway damage, and long-standing asthma [41]. They are chronic inflammatory diseases characterized by an imbalance between oxidative processes and pulmonary antioxidant mechanisms [42].

A higher intake of flavonoids, including quercetin, is associated with a lower risk of developing COPD, especially in current and former smokers. High intake reduced the risk by up to 20% compared with lower levels. Although flavonoids offer a partial protective effect, smoking cessation remains the main priority [43]. In addition, in a clinical study, the safety of quercetin was evaluated in patients with mild-to-severe COPD by administering increasing doses, including (500, 1000, and 2000 mg/day) for one week. No serious adverse events or alterations in hematological or biochemical parameters were recorded. These results indicate that quercetin is well tolerated and safe at concentrations of up to 2000 mg/day in this population [44].

Acute exacerbations are a significant cause of morbidity and mortality in COPD patients, and rhinovirus plays a key role in this process. In a murine model with a COPD phenotype, rhinovirus infection-induced lung inflammation, goblet cell metaplasia, increased cholinergic airway responsiveness, and immune cell accumulation were not observed in healthy mice. Quercetin supplementation significantly attenuated these pathological effects, including pulmonary mechanical dysfunction [45].

COPD is a leading cause of morbidity and mortality worldwide, with cigarette smoke being a key factor in its development. In a murine model, the protective effect of quercetin against prolonged exposure to cigarette smoke was evaluated. Mice treated with 10 mg/kg/day quercetin presented reduced cellular infiltration, oxidative stress, and lung inflammation. In addition, they demonstrated improvements in histological structure and a reduction in emphysema compared with the untreated group [46].

Moreover, corticosteroid resistance represents a significant limitation in the treatment of COPD, which is induced in part by oxidative stress and chronic inflammation. A study demonstrated that quercetin restores corticosteroid sensitivity in human cells exposed to cigarette smoke extract and in immune cells from COPD patients by antioxidant and AMPK-activating properties. In the presence of quercetin, the AMPK/Nrf2 pathway was activated, reversing the induced insensitivity and improving the efficacy of dexamethasone [47]. Furthermore, one study explored the underlying mechanism by which Jinwei decoction enhances the anti-inflammatory effect of glucocorticoids (GCs) in COPD, focusing on the regulation of HDAC2 expression through miR-155-5p. By network pharmacology, GO and KEGG enrichment analyses, in an in vitro study with BEAS-2B cells exposed to cigarette smoke extract, 127 genes common to COPD, Jinwei, and miR-155-5p were identified. The results revealed that the active compounds quercetin, luteolin, and stigmasterol have high affinities for key proteins, including PTGS2, HIF1A, and AKT1. Jinwei decoction reversed miR-155-5p overexpression, reduced PI3K‒Akt pathway activation, and increased HDAC2 levels, thus enhancing the anti-inflammatory response to GC [48].

Finally, in the context of COPD, miRNAs, such as miR-155, play key roles in regulating the inflammatory response and the progression of lung damage. Studies have shown that miR-155 is overexpressed in the immune cells of COPD patients and promotes the production of proinflammatory cytokines, such as TNF-α, IL-1β, and IL-6, as well as the activation of the NF-κB pathway, contributing to the chronic inflammatory environment characteristic of this disease [49]. In this context, quercetin has been shown to significantly reduce miR-155 levels in activated macrophages, an effect accompanied by the inhibition of inflammatory signaling and an increase in the activity of the antioxidant factor Nrf2. This dual regulation of miR-155 and Nrf2 by quercetin suggests a mechanism by which the balance between proinflammatory and antioxidant processes in damaged lung tissue can be restored. Thus, the modulation of miR-155 by quercetin represents a promising therapeutic avenue to reduce persistent inflammation and functional impairment in COPD patients [50].

These findings suggest that quercetin could be a promising adjunctive therapy in COPD patients to treat corticosteroid resistance and viral exacerbations, such as rhinovirus, slow disease progression, and prevent cigarette smoke-induced emphysema (Table 1).

#### 5.1.2. Quercetin and Lung Cancer

LC is a comorbidity of IPF that is a significant predictor of survival and has a prevalence of 16% in people diagnosed with IPF, where 19% are small-cell LC and 81% are non-small-cell lung cancer (NSCLC) [1]. LC is the leading cause of cancer mortality globally, with an incidence of approximately 2.3 million new cases and approximately 1.8 million deaths each year [2].

LC, especially NSCLC, is associated with chronic inflammatory processes, oxidative stress, and epigenetic alterations that facilitate tumor proliferation and survival. Epidemiological studies have revealed that a greater intake of quercetin-rich foods is associated with a lower risk of LC, a finding supported by a population-based case‒control study in Italy. Quercetin exerts chemopreventive effects through free radical scavenging, activation of phase II enzymes, induction of apoptosis, and modulation of antiproliferative and inflammatory pathways [3].

At the molecular level, a diet rich in quercetin has been shown to modulate the expression of functional miRNAs in lung tissue, including members of the let-7, miR-146, miR-26, and miR-17 families, with significant changes, especially in the adenocarcinomas of ex-smokers. In total, 48 differentially expressed miRNAs involved in metastasis suppression, proliferation, and apoptosis induction, including miR-125a, miR-503, miR-16, and let-7, were identified. These findings suggest that quercetin can modulate lung carcinogenesis through epigenetic regulation of key miRNAs, particularly in individuals with prior exposure to smoking [51].

In addition, quercetin has been shown to increase the radiosensitivity of NSCLC cells in a dose- and time-dependent manner, an effect mediated by the regulation of the miR-16-5p/WEE1 axis. Quercetin treatment induces the overexpression of miR-16-5p and the repression of WEE1, [52] a key G2/M checkpoint tyrosine kinase involved in the DNA damage response. WEE1 is upregulated in multiple malignancies, including NSCLC, and its elevated expression is associated with resistance to radiotherapy [53]. The miR-15 family, comprising miR-15a, miR-15b, and miR-16, has been described as a modulator of radiosensitivity in various solid tumors. In particular, miR-16-5p potentiates the response to radiation in NSCLC [54].

Finally, quercetin reduces claudin-2 expression in A549 cells by decreasing the stability of claudin-2 mRNA without affecting the promoter activity of the gene. This effect is associated with the specific upregulation of miR-16, whose inhibition reverses the quercetin-induced decrease in claudin-2 [55]. MiR-16, which is frequently repressed in NSCLC tissues, acts as a tumor suppressor by inhibiting cell proliferation, migration, and invasion [56].

These findings suggest that quercetin could be a promising adjunctive therapy in lung cancer, particularly non-small-cell lung cancer (NSCLC), by modulating epigenetic regulators such as microRNAs involved in tumor suppression, enhancing radiosensitivity, and inhibiting tumor cell proliferation, migration, and chemoresistance (Table 2).

#### 5.1.3. Quercetin and Pulmonary Hypertension

PH is a disease characterized by pulmonary vasoconstriction, proliferation of vascular smooth muscle cells, and remodeling of the pulmonary arteries. It is one of the comorbidities presented by people with IPF, with a prevalence ranging from 14 to 84% [57].

Quercetin has shown promising therapeutic potential by targeting key molecular pathways involved in PH pathogenesis [58]. In experimental models, quercetin regulates the expression of noncoding RNAs relevant to PH, such as long noncoding RNAs (lncRNA) H19, myocardial infarction-associated transcript (MIAT), and miR-29a and miR-33a, which are involved in cell proliferation and apoptosis in pulmonary vascular tissue. By influencing these molecules, quercetin attenuates vascular smooth muscle hyperplasia and fibrosis, improving pulmonary function in monocrotaline-induced PH rats [59,60].

In addition, quercetin induces apoptosis and autophagy in pulmonary artery smooth muscle cells (PASMCs) under hypoxic conditions both in vitro and in vivo. In this study, it was observed to increase the expression and activity of FOXO1, whose inhibition blocks quercetin-induced autophagy by interfering with the mTOR/4E-BP1 pathway. Inhibition of autophagy sensitizes cells to quercetin-induced apoptosis [61].

Furthermore, quercetin reverses excessive proliferation and resistance to hypoxia-induced apoptosis in PASMCs. It inhibits cell proliferation and migration, induces apoptosis and arrest at the G0/G1 phase, and regulates the expression of cyclins and migration- and survival-related proteins. These effects are related to the dose-dependent inhibition of the TrkA/AKT pathway. In addition, it modulates the Bax/Bcl-2 ratio and decreases the expression of MMP2, MMP9, CXCR4, and integrins [62].

Finally, recent studies have shown that quercetin blocks the proliferation, migration, and phenotypic transformation of human HPASMCs by inhibiting the TGF-β1/Smad2/Smad3 pathway. This action contributes to attenuating right ventricular hypertrophy and pulmonary vascular remodeling, supporting its potential as a therapeutic agent at the pharmacological and molecular levels for the treatment of PH [63].

These findings demonstrate that quercetin modulates inflammatory and oxidative pathways and preserves cellular homeostasis, underscoring its pharmacological and molecular potential in PAH treatment (Table 3).

### 5.2. Extrapulmonary Comorbidities

#### 5.2.1. Quercetin and Cardiovascular Disease

CVDs represent one of the leading causes of morbidity and mortality worldwide, with oxidative stress and chronic inflammation playing a central role in their pathogenesis. Among them, CAD stands out as one of the most prevalent, with an incidence of 77.9% in patients with IPF [7,64,65].

Several preclinical studies have demonstrated that quercetin has significant cardioprotective effects by modulating gene pathways associated with oxidative stress, inflammation, and endothelial dysfunction. In a study, the consumption of foods rich in quercetin was associated with lower mortality from CAD [66]. Similarly, in patients with stable ischemic heart disease treated with quercetin, decreased expression of IL-1β, TNF-α, and IκBα was observed, suggesting decreased NF-κB activity and supporting the anti-inflammatory potential of this flavonoid in CVD [67]. Both IL-1β and TNF-α are key mediators of the inflammatory response, contributing to endothelial dysfunction and vascular damage by inducing their synthesis through the transcriptional activation of NF-κB [68].

On the other hand, quercetin reportedly protects human coronary artery endothelial cells against hypoxia/reoxygenation (H/R)-induced damage by mitigating oxidative stress and mitochondrial apoptosis. This effect is associated with a reduction in the production of reactive oxygen species (ROS), an increase in antioxidant activity including superoxide dismutase (SOD) and catalase (CAT), and the activation of the Nrf2/HO-1 pathway, thus strengthening the cellular defense against oxidative damage. Furthermore, dose-dependent inhibition of the expression of active caspase-3, a marker of apoptosis, was observed [69].

Additionally, hyperoside (quercetin-3-O-galactoside), a natural derivative of quercetin, has been shown to reduce miR-21 levels, promote cell viability, and suppress the inflammatory response, which attenuates sepsis-induced cardiac dysfunction [70]. Since miR-21 is also implicated in fibrosis and cardiac remodeling, its inhibition by compounds such as quercetin is relevant in models of heart failure [71].

Ultimately, quercetin may enhance the beneficial effects of physical exercise by modulating the expression of miRNAs associated with CVD. In a study conducted in LDL-/- mice fed an atherogenic diet, the combination of exercise and quercetin upregulated the expression of miR-21 and miR-125b [72]. Notably, miR-21 has both atheroprotective and proinflammatory effects, depending on the pathophysiological context [73]; miR-451 is associated with apoptotic processes and tumor biomarkers [74]; and miR-125b, which is abundant in the vascular system, is involved in regulating cell damage and inflammation [75].

Taken together, these findings suggest that quercetin, either alone or in combination with exercise, acts synergistically on key molecular pathways involved in inflammation, oxidative stress, and structural remodeling of the heart, thereby reinforcing its potential as a cardioprotective agent in the context of CVD (Table 4).

#### 5.2.2. Quercetin and Diabetes

Diabetes mellitus (DM) is a common comorbidity in patients with IPF and is associated with an increased risk of disease progression and an increased mortality rate [76,77,78]. A recent meta-analysis suggested that people with IPF have a 1.54-fold increased risk of developing DM compared with individuals without IPF [79]. Epidemiological studies have estimated that between 10 and 42% of patients diagnosed with IPF have DM as a comorbidity [1,76]. On the other hand, a recent meta-analysis indicated that approximately 16% of patients with IPF present with DM as a comorbidity [8]. This coexistence may be attributed, in part, to the chronic inflammatory state, oxidative stress, and metabolic dysfunction shared by both pathologies [76,77,78].

In this context, quercetin has demonstrated relevant antidiabetic effects; several investigations in murine models of T2DM have shown that quercetin has a consistent hypoglycemic effect [80,81]. In C57BL/KsJ-db/db mice receiving quercetin-enriched diets, a significant decrease in plasma glucose was recorded, and a dose-dependent effect was observed. This decrease was accompanied by an improvement in HOMA-IR index values without any substantial changes in insulin concentrations, indicating a possible improvement in treatment-induced insulin sensitivity [81]. On the other hand, a second study confirmed that quercetin decreased fasting glucose levels in diabetic mice while increasing glucose transporter (GLUT) 4 expression [80]. In both models, complementary benefits, such as reduced oxidative stress, improved lipid profiles, and decreased DNA damage, were also recorded, reinforcing the idea that quercetin not only improves hyperglycemia but also exerts a comprehensive protective effect in the context of T2DM [80,81].

Several studies have shown that quercetin has anti-diabetic effects primarily through the activation of the AMPK pathway, a key regulator of cellular energy metabolism. This mechanism promotes glucose uptake in muscle cells by promoting the expression and translocation of the GLUT4 transporter to the plasma membrane, independent of insulin [82,83]. Furthermore, in hepatocytes, AMPK activation by quercetin inhibits the expression of gluconeogenic enzymes, such as glucose-6-phosphatase (G6 Pase), thereby reducing hepatic glucose production [82]. Additionally, quercetin can increase the AMP/ATP ratio, transiently alter the mitochondrial potential, and increase the intracellular calcium level, suggesting the possible involvement of calcium/calmodulin-mediated protein kinases (CaMKKs) in AMPK activation [83].

In parallel, quercetin exerts antioxidant and anti-inflammatory effects by suppressing the expression of genes associated with oxidative stress and systemic inflammation [80,84,85]. For example, quercetin was shown to negatively regulate key genes related to ROS production in Achilles tendon-derived cells from hyperglycemic Sprague‒Dawley rats. In particular, it significantly decreased the expression of the key Nox1 and Nox4 enzymes involved in ROS production. It also reduces the expression of Il6, a crucial proinflammatory mediator [84]. Moreover, a study in primary cultures of human adipocytes demonstrated that quercetin exerts a significant anti-inflammatory effect by decreasing the gene expression and secretion of proinflammatory cytokines, such as IL-6, IL-1β, IL-8, and MCP-1. At the intracellular signaling level, quercetin inhibited the activation of the ERK, JNK, and NF-κB pathways, which are commonly stimulated by TNF-α, thus limiting the inflammatory response. It also prevents the reduction in the expression and transcriptional activity of the nuclear factor PPARγ, as well as its target genes, keeping them active despite the proinflammatory environment. With respect to insulin resistance, quercetin prevents serine phosphorylation of IRS-1 and reduces PTP-1B expression, leading to improved insulin-dependent glucose uptake [85]. Together, these results suggest that this gene regulation contributes to lowering insulin resistance induced by increased levels of ROS and proinflammatory cytokines, a central mechanism in the pathophysiology of T2DM [80,84,85].

Additionally, quercetin can modulate the expression of miRNAs involved in the development of DM2, including miR-92b-3p, miR-485-5p, miR-29a, miR-29b, and miR-29c, suggesting a relevant epigenetic role [86,87,88]. In a murine model of T2DM, quercetin was shown to exert antidiabetic effects through modulation of the miR-92b-3p/EGR1 axis. Quercetin treatment increased the expression of miR-92b-3p and reduced that of EGR1, a metabolic and inflammatory stress-induced transcription factor whose overexpression under hyperglycemic conditions has been associated with insulin resistance, chronic inflammation, and pancreatic damage. EGR1 promotes the transcription of genes linked to proinflammatory pathways and the functional impairment of β-cells. In this context, miR-92b-3p acts as a negative regulator of EGR1, so its restoration by quercetin represents a relevant mechanism to inhibit this pathological pathway and attenuate the alterations associated with T2DM [86]. In a murine model of streptozotocin-induced diabetes, quercetin administration significantly reduced the expression of members of the miR-29 family, including miR-29a, miR-29b, and miR-29c, in the hippocampus. This family of miRNAs is markedly overexpressed in diabetic rats, and its activation is related to the repression of key genes involved in glucose metabolism, such as GLUT1, GLUT2, GLUT3, and GLUT4 transporters, as well as insulin-like growth factor 1 (IGF-1). Quercetin treatment reversed this dysregulation by decreasing miR-29 expression, thereby restoring the levels of GLUTs and IGF-1. These effects suggest that quercetin improves glycemic homeostasis in the central nervous system through posttranscriptional modulation of the miR-29/GLUT/IGF-1 axis, which may represent a promising therapeutic strategy for preventing neurological complications associated with diabetes [87].

Furthermore, in an in vitro model of high glucose-induced diabetic nephropathy in human mesangial cells (HMCs), quercetin was shown to attenuate proliferation, inflammation, and oxidative stress by regulating the miR-485-5p/YAP1 axis. The expression of miR-485-5p is decreased in both patients with diabetic nephropathy and HMCs exposed to high glucose. This miRNA acts as a direct inhibitor of YAP1, a transcriptional coactivator implicated in the progression of kidney damage. Quercetin treatment of HMCs exposed to high glucose increased miR-485-5p and reduced YAP1 expression, which in turn decreased the levels of proinflammatory cytokines, such as TNF-α, IL-1β, and IL-6, and lipid peroxidation (MDA) and improved the activity of antioxidant enzymes such as SOD and GSH-Px. These results suggest that modulation of the miR-485-5p/YAP1 axis constitutes a relevant mechanism by which quercetin exerts protective effects against the renal complications of diabetes [88]. The ability of quercetin to modulate diverse molecular mechanisms holds promise not only for the control of T2DM but also for the treatment of associated complications and comorbidities, such as diabetic nephropathy and oxidative damage in peripheral tissues. By reducing the proinflammatory state, improving insulin sensitivity, and restoring redox balance, quercetin could indirectly contribute to attenuating the progression of chronic multisystem diseases [80,81,84,85,86,87,88].

These findings suggest that quercetin could be a promising adjunctive therapy for type 2 diabetes mellitus by improving glycemic control, enhancing insulin sensitivity, and regulating oxidative stress and chronic inflammation (Table 5).

#### 5.2.3. Quercetin and Psychiatric Diseases

Anxiety and depression are associated with significant increases in oxidative stress and neuroinflammation, which contribute to neuronal dysfunction and alterations in the hypothalamic‒pituitary‒adrenal (HPA) axis. These disorders represent frequent comorbidities in patients with IPF, with a prevalence of 25.9% for anxiety and 21.4% for depression. Both symptoms negatively impact quality of life and complicate clinical management [89,90,91,92].

Several studies have demonstrated the therapeutic potential of quercetin as an anxiolytic and antidepressant agent that acts on multiple molecular pathways involved in chronic stress. In murine models, quercetin has been shown to reverse the anxiogenic and depressive effects induced by corticotropin-releasing factor (CRF), as evidenced by reduced immobility time in the forced swim test and increased social interaction time. These effects are comparable to those induced by classic drugs such as fluoxetine or diazepam and appear to be mediated by modulation of the HPA axis, which plays a central role in the pathophysiology of emotional stress [93].

From a perspective focused on neuroinflammation and mitochondrial dysfunction, quercetin has been shown to attenuate methamphetamine (MA)-induced anxious behavior by improving mitochondrial bioenergetics and reducing oxidative stress. In animal models, its administration significantly decreased ROS levels, improved the mitochondrial membrane potential, and increased ATP production. Furthermore, it inhibited astrocyte activation and reduced the expression of proinflammatory cytokines such as IL-1β and TNF-α. However, it had no significant effect on IL-6, suggesting that it has a selective effect on specific inflammatory pathways [94].

At the systemic and brain levels, depression is characterized by sustained activation of inflammatory pathways, oxidative stress, and mitochondrial dysfunction. An increase in lipid peroxidation biomarkers, such as MDA, and a decrease in the activity of antioxidant enzymes, including SOD and CAT, have been documented. In this context, polyphenols—and particularly quercetin—have shown significant neuroprotective, antioxidant, and anti-inflammatory effects. Preclinical studies support their usefulness as adjuvants in the dietary treatment of depressive disorders by improving brain redox status and promoting synaptic plasticity [95].

Adult neurogenesis in the hippocampus, especially in the dentate gyrus, plays a crucial role in recovery from depressive symptoms. In a mouse model of depression induced by chronic mild and unpredictable stress, quercetin administration significantly improved depressive behavior and promoted the restoration of hippocampal neurogenesis. This effect is associated with the inhibition of microglia-derived exosomes carrying the miRNA let-7e-5p, which negatively affects neural stem cells by interfering with the Wnt1/β-catenin signaling pathway. Coculture studies and luciferase assays confirmed that let-7e-5p directly regulates Wnt1 expression, thereby limiting neuronal proliferation [96].

On the other hand, isoflurane anesthesia has been shown to induce cognitive dysfunction, impairing postoperative recovery. In an anesthetized mouse model, quercetin treatment improved memory and reduced neuroinflammation in the hippocampus. This effect was associated with an increase in the expression of miR-138-5p, which downregulates the LCN2 gene, a factor linked to inflammatory processes. Inhibition of miR-138-5p abrogated the beneficial effects of quercetin, increasing the levels of TNF-α, IL-1β, and IL-6. These findings suggest that quercetin protects against anesthesia-induced cognitive impairment by modulating the miR-138-5p/LCN2 pathway [97].

Finally, quercetin has been shown to protect PC-12 neuronal cells from hydrogen peroxide-induced damage. In this study, an oxidative stress model was used to investigate the molecular effects of quercetin by sequencing lncRNAs, miRNAs, and mRNAs. In total 297 lncRNAs, 194 miRNAs, and 14 mRNAs were identified as significantly repressed after treatment, suggesting complex epigenetic regulation. Bioinformatics analysis indicated that the PI3K/AKT pathway plays a key role in the protective effects of quercetin. Furthermore, an endogenous competitor RNA (ceRNA) network was constructed, providing new insights into the molecular mechanisms of oxidative damage and the neuroprotective potential of this compound [98].

These findings suggest that quercetin could be a promising adjunctive therapy for psychiatric disorders, particularly anxiety, depression, and stress-related cognitive dysfunction, through its antioxidant, anti-inflammatory, and neuroprotective effects (Table 6).

## 6. Conclusions and Future Directions

IPF represents a progressive and lethal disorder whose clinical complexity is compounded by the presence of multiple pulmonary and systemic comorbidities. Despite therapeutic advances, current treatments remain limited and do not offer an effective reversal of fibrosing damage.

**Figure 4:**
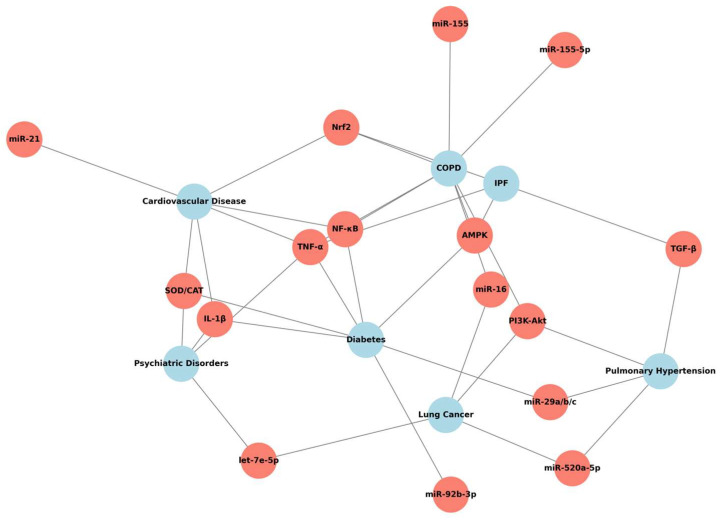
Quercetin targeting key molecular pathways of biomarker networks involved in IPF and comorbidities. Red circles represent proteins and miRNAs modulated by quercetin which are involved in IPF, and its comorbidities are represented in blue circles.

In this context, miRNAs have emerged as key regulators of gene expression in processes related to fibrosis, inflammation, senescence, and tissue remodeling. In IPF and its main comorbidities COPD, PH, LC, diabetes, CVD, and neuropsychiatric disorders, substantial alterations in the expression of miRNAs, such as miR-21, miR-16, miR-155, miR-29, miR-92b-3p, and let-7, among others, have been demonstrated (Figure 4).

Quercetin, a naturally occurring flavonoid, has demonstrated not only antioxidant and antifibrotic effects but also a remarkable ability to modulate the profile of dysfunctional miRNAs in multiple preclinical models and pilot studies in humans. Its ability to restore redox homeostasis and inhibit proinflammatory pathways, such as the NF-κB, TGF-β/Smad, PI3K/AKT, and TrkA pathways, is directly linked to the modulation of profibrotic and proinflammatory miRNAs, such as the repression of miR-155 and miR-29 or the induction of miR-16, miR-92b-3p, and miR-138-5p.

These findings position quercetin as an agent with multifaceted therapeutic potential that is capable of interfering with molecular mechanisms common to several chronic diseases. Its impact on post-transcriptional epigenetic regulation via miRNAs suggests not only a symptomatic benefit but also an opportunity to modify the pathological course of IPF and its associated comorbidities.

However, significant challenges remain, such as its limited oral bioavailability—due to its low water solubility, rapid metabolism and limited intestinal permeability—which represents a major limitation for its clinical application, but could be overcome by advanced pharmaceutical strategies among them include: Nanoparticle encapsulation [99], liposomal formulations [100], prodrug approaches, cyclodextrin complexes [101], and Co-administration with absorption enhancers [102].

Despite the urgent need for enhanced antifibrotic therapies, no published clinical studies have directly assessed the combination of quercetin with pirfenidone or nintedanib. However, this is a promising direction. Mechanistically, these compounds act on complementary fibrotic and inflammatory pathways.

Taken together, this evidence strongly supports the need to continue investigating quercetin in clinical settings, particularly in models focused on miRNA networks, to develop targeted and more effective therapies for patients with IPF and associated diseases.

Overall, these findings position quercetin as a bioactive agent with the ability to modulate gene expression and miRNAs involved in the progression of multiple comorbidities associated with IPF, not as a therapeutic substitute but as an adjuvant tool that could be integrated within a multimodal and personalized therapeutic approach.

## Figures and Tables

**Table 1:** Reported therapeutic effects of quercetin in COPD models.

Model/Study	Molecules or Pathways Regulated	Observed Effect	Therapeutic Category	Type of Evidence	Reference
**Murine model of COPD exposed to rhinovirus + quercetin**	↓ inflammation, ↓ goblet cell metaplasia, ↓ cholinergic response	↓ airway inflammation and dysfunction	Anti-inflammatory	In vivo	[45]
**Murine model of COPD induced by cigarette smoke + quercetin**	↓ cellular infiltration, ↓ IL-10, IL-13 e IL-22, ↑ SOD, CAT	↓ lung inflammation and tissue damage, ↓ oxidative stress, ↓ emphysema	Anti-inflammatory, Antioxidant Immunomodulator	In vivo	[46]
**Human monocytic U937 cells + cigarette smoke extract + quercetin** **Human PBMCs from COPD patients + quercetin**	↑ AMPK/Nrf2, ↓ corticosteroid resistance	↑ steroid sensitivity, ↓ oxidative stress	Antioxidant, Corticosteroid Sensitizer	In vitro	[47]
**BEAS-2B cells + cigarette smoke extract + Jinwei decoction (quercetin, luteolin, stigmasterol)**	↓ miR-155-5p, ↓ PI3K-Akt, ↑ HDAC2	↓ inflammation, ↑ glucocorticoid response	Anti-inflammatory, Epigenetic Modulator	In vitro	[48]
**LPS-activated macrophages + quercetin**	↓ miR-155, ↑ Nrf2/HO-1	↓ NF-κB, iNOS, TNF-α, IL1β, and IL-6	Anti-inflammatory, Antioxidant	In vitro	[50]

The symbol “↓” indicates downregulation, and “↑” indicates upregulation. COPD, chronic obstructive pulmonary disease, PBMCs, peripheral blood mononuclear cells: LPS, lipopolysaccharide.

**Table 2:** Reported therapeutic effects of quercetin in lung cancer models.

Model/Study	Molecules or Pathways Regulated	Observed Effect	Therapeutic Category	Type of Evidence	Reference
**GLC-82 and HTB-182 cell lines + quercetin treatment**	↑ miR-16-5p, ↓ WEE1	↑ radiosensitivity	Radiosensitizer	In vitro	[52]
**A549 + quercetin treatment**	↑ miR-16, ↓ claudin-2	↓ proliferation, ↓ migration ↓ invasión	Antitumor, Antimetastatic	In vitro	[55]
**Lung cancer tissue after quercetin-rich diet**	↑ miR-let-7, miR-146, miR-26, miR-17, miR-125a, miR-503, miR-16	↓ proliferation, ↑ apoptosis	Chemopreventive, Epigenetic modulator	In vivo	[51]

The symbol “↓” indicates downregulation, and “↑” indicates upregulation. NSCLC, non-small-cell lung cancer.

**Table 3:** Reported therapeutic effects of quercetin in pulmonary hypertension models.

Model/Study	Molecules or Pathways Regulated	Observed Effect	Therapeutic Category	Type of Evidence	Reference
**MCT-induced PH rats + quercetin**	↓ lncRNA H19, ↓ MIAT, ↓ miR-29a/33a	↓ vascular smooth muscle cell proliferation and fibrosis	Antiproliferative, Antifibrotic, Epigenetic Modulator	In vivo	[60]
**MCT-induced PH rats + quercetin**	↑ miR-204, ↓ PARP1, ↓ HIF1α, ↓ NFATc2, ↓ α-SMA	↓ pulmonary artery pressure, ↓ vascular remodeling, and inflammation	Anti-inflammatory, Antiproliferative, Epigenetic Modulator	In vivo	[59]
**Hypoxia-induced PASMCs + quercetin**	↑ LC3-II, Beclin-1, Atg5, SESN3, FOXO1; ↓ p-mTOR, ↓ 4E-BP1, ↓ p70S6K	↑ autophagy, ↑ apoptosis; inhibition of mTOR pathway; FOXO1-dependent mechanism	Pro-autophagic, Pro-apoptotic	In vitro	[61]
**Hypoxia-induced PH in rats + quercetin**	↑ FOXO1, ↑ LC3-II, ↑ apoptosis; ↓ RVSP, ↓ vascular remodeling	↓ pulmonary artery pressure and wall thickness; ↑ lung cell apoptosis	Antiproliferative, Pro-Apoptotic	In vivo	[61]
**Hypoxia-induced PH in rats + quercetin**	↓ RVSP, ↓ RV/LV+S, ↓ vascular remodeling, ↓ PCNA/Ki67, ↑ apoptosis, ↓ p-TrkA, ↓ p-AKT	↓ proliferation and remodeling, ↑ apoptosis in PASMCs	Antiproliferative, Pro-Apoptotic	In vivo	[62]
**Hypoxia-induced PASMCs + quercetin**	↓ MMP2/9, ↓ CXCR4, ↓ integrins α5/β1, ↓ p-TrkA, ↓ p-AKT, ↑ Bax/Bcl-2	↓ proliferation and migration, ↑ apoptosis	Antiproliferative, Pro-Apoptotic	In vitro	[62]
**HPASMCs + PDGF-BB + quercetin**	↓ PCNA, ↓ OPN, ↓ TGF-β1, ↓ p-Smad2/3, ↑ α-SMA	↓ cell proliferation and migration; ↑ apoptosis; ↓ phenotypic switching	Antiproliferative, Antifibrotic	In vitro	[63]
**MCT-induced PH rats + quercetin**	↓ PCNA, ↓ OPN, ↓ TGF-β1, ↓ p-Smad2/3, ↑ α-SMA	↓ pulmonary artery pressure and wall thickness (mPAP, WT%, WA%), ↓ RV hypertrophy index, ↓ vascular remodeling	Antiproliferative, Antifibrotic	In vivo	[63]

The symbol “↓” indicates downregulation, and “↑” indicates upregulation. MCT, monocrotaline: PASMCs, smooth muscle cells of the pulmonary artery: PH, pulmonary hypertension: HPASMCs, human smooth muscle cells of the pulmonary artery: PDGF-BB, platelet-derived growth factor BB.

**Table 4:** Reported therapeutic effects of quercetin in cardiovascular disease models.

Model/Study	Molecules or Pathways Regulated	Observed Effect	Therapeutic Category	Type of Evidence	Reference
**Stable CAD patients + quercetin**	↓ IL-1β, ↓ TNF-α, ↓ IκBα ↓ NF-κB	↓ inflammatory signaling	Anti-inflammatory	Clinical	[67]
**CAECs + H/R + quercetin**	↓ ROS, ↑ SOD, ↑ CAT, ↑ Nrf2/HO-1, ↓ caspase-3	↓ oxidative damage, ↓ apoptosis, ↑ antioxidant	Antioxidant, Antiapoptotic	In vitro	[69]
**Mice (LDL^−^/^−^ + atherogenic diet) + quercetin + exercise**	↑ miR-21, ↑ miR-125b	Modulation of miRNAs associated with vascular health	Epigenetic Modulator, Cardioprotective	In vivo	[72]
**Sepsis-induced cardiac dysfunction + hyperoside**	↓ miR-21, ↓ IL-6, ↓ TNF-α, ↓ cTnI, ↓ CK-MB	↓ inflammation, improved cardiac function	Anti-inflammatory, Epigenetic Modulator	In vivo	[70]
**H9C2 cell line + LPS + hyperoside**	↓ miR-21, ↓ IL-6, ↓ TNF-α ↑ cell viability	↓ inflammation	Anti-inflammatory, Epigenetic Modulator	In vitro	[70]

The symbol “↓” indicates downregulation, and “↑” indicates upregulation. CAD, coronary artery disease: CAECs, coronary artery endothelial cells: H/R, hypoxia/reoxygenation: LPS, lipopolysaccharide: hyperoside, quercetin-3-O-galactoside.

**Table 5:** Reported therapeutic effects of quercetin in diabetes models.

Model/Study	Molecules or Pathways Regulated	Observed Effect	Therapeutic Category	Type of Evidence	Reference
**C57BL/KsJ-db/db mice with quercetin-enriched diet**	↓ plasma glucose, ↓ HOMA-IR, ↑ adiponectin, ↓ TG, ↓ CT, ↑ HDL, ↓ TBARS, ↑ SOD/CAT/GSH-Px	↓ hyperglycemia, ↓ dyslipidemia, ↓ oxidative stress, ↑ insulin sensitivity	Hypoglycemic, Antioxidant, Insulin Sensitizer	In vivo	[81]
**Model of DM induced by aloxane + quercetin**	↓ fasting blood glucose, ↑ GLUT4, ↑ GSH, ↑ SOD, ↑ CAT, ↓ TBARS, ↓ DNA damage	↓ hyperglycemia, ↓ oxidative stress, ↓ cytotoxicity	Hypoglycemic, Antioxidant	In vivo	[80]
**L6 skeletal muscle cells, H4IIE and HepG2 cell lines**	↑ AMPK, ↑ GLUT4 translocation, ↓ G6Pase.	↑ muscle glucose uptake, ↓ hepatic gluconeogenesis	Hypoglycemic, Insulin Sensitizer	In vitro	[82,83]
**Achilles tendon cells from diabetic rats + quercetin**	↓ Nox1, ↓ Nox4, ↓ Il6	↓ ROS, ↓ inflammation, ↓ cell apoptosis.	Antioxidant, Anti-inflammatory	In vitro	[84]
**Primary human adipocytes treated with TNF-α + quercetin**	↓ IL-6, ↓ IL-1β, ↓ IL-1β, ↓ IL-8, ↓ MCP-1, ↓ ERK/JNK/NF-κB, ↓ PTP-1B, ↓ p-IRS-1(Ser), ↑ PPARγ	↓ inflammation, ↓ insulin resistance, ↑ glucose uptake	Anti-inflammatory, Insulin Sensitizer	In vitro	[85]
**DM model induced by high-fat/-glucose diet and STZ + quercetin**	↑ miR-92b-3p, ↓ EGR1	↓ inflammation, ↓ insulin resistance, ↓ pancreatic damage	Epigenetic Modulator, Anti-inflammatory	In vivo	[86]
**Model of DM induced by STZ + quercetin**	↓ miR-29a/b/c, ↑ GLUT1-4, ↑ IGF-1.	↑ cerebral glucose metabolism, ↓ neurological alterations	Epigenetic Modulator, Neuroprotector	In vivo	[87]
**HMCs + HG + quercetin**	↑ miR-485-5p, ↓YAP1, ↓ YAP1, ↓ TNF-α, ↓ IL-1β, ↓ IL-6, ↓ MDA, ↑ SOD, ↑ GSH-Px	↓ proliferation, ↓ inflammation, ↓ oxidative stress, ↑ antioxidant function	Epigenetic Modulator, Antioxidant, Anti-inflammatory	In vitro	[88]

The symbol “↓” indicates downregulation, and “↑” indicates upregulation. DM, diabetes mellitus; STZ, streptozotocin; HG, high glucose; HMCs, human mesangial cells.

**Table 6:** Reported therapeutic effects of quercetin in psychiatric disease models.

Model/Study	Molecules or Pathways Regulated	Observed Effect	Therapeutic Category	Type of Evidence	Reference
**CRF-induced stress in mice + quercetin**	Modulation of HPA axis	↓ anxiety and depression-like behavior (comparable to fluoxetine/diazepam)	Anxiolytic, Antidepressant	In vivo	[93]
**MA-induced anxiety in mice + quercetin**	↓ ROS, ↑ mitochondrial potential, ↑ ATP, ↓ IL-1β, ↓ TNF-α, ↓ astrocyte activation	↓ anxiety-like behavior, ↓ neuroinflammation, ↑ mitochondrial function	Antioxidant, Neuroprotective, Anti-inflammatory	In vivo	[94]
**CUMS-induced depression in mice + quercetin**	↑ neurogenesis (↑ Wnt1/β-catenin), ↓ let-7e-5p from microglial exosomes	↓ depressive behavior, ↑ hippocampal neurogenesis	Antidepressant, Neurogenic, Epigenetic Modulator	In vivo	[96]
**ISO-induced cognitive impairment in rats + quercetin**	↑ miR-138-5p, ↓ LCN2, ↓ TNF-α, ↓ IL-1β, ↓ IL-6	↓ cognitive impairment, ↓ neuroinflammation	Neuroprotective, Epigenetic Modulator	In vivo	[97]
**PC12 cells + H_2_O_2_ + quercetin**	297 lncRNAs, 194 miRNAs, and 14 mRNAs dysregulated	↓ oxidative damage, regulation of ceRNA network	Antioxidant, Epigenetic Modulator	In vitro	[98]

The symbol “↓” indicates downregulation, and “↑” indicates upregulation. HPA, hypothalamus–pituitary–adrenal: CRF, corticotrophin-releasing factor: MA, methamphetamine: CUMS, chronic unpredictable mild stress: ISO, Isoflurane.

## Data Availability

Not applicable.

## Abbreviations

The following abbreviations are used in this manuscript:

IPF	Idiopathic Pulmonary Fibrosis
COPD	Chronic Obstructive Pulmonary Disease
PH	Pulmonary Hypertension
LC	Lung Cancer
CVDs	Cardiovascular Diseases
CAD	Coronary Artery Disease
FDA	Food and Drug Administration
TGF-β	Transforming Growth Factor Beta
TNF-α	Tumor Necrosis Factor-alpha
PDGF	Platelet-Derived Growth Factor
FGF	Fibroblast Growth Factor
VEGF	Vascular Endothelial Growth Factor
miRNAs	microRNAs
ECM	Extracellular Matrix
EGCG	Epigallocatechin Gallate
SASP	Senescence-Associated Secretory Phenotypes
BLM	Bleomycin
AMPK	AMP-activated protein kinase
GCs	Glucocorticoids
NSCLC	Non-Small-Cell Lung Cancer
MIAT	Myocardial Infarction-Associated Transcript
PASMCs	Pulmonary Artery Smooth Muscle Cells
H/R	Hypoxia/Reoxygenation
ROS	Reactive Oxygen Species
SOD	Superoxide Dismutase
CAT	Catalase
DM	Diabetes Mellitus
GLUT	Glucose Transporter
G6 Pase	Glucose-6-phosphatase
CaMKKs	Calcium/Calmodulin-Mediated Protein Kinases
IGF-1	Insulin-Like Growth Factor 1
HMCs	Human Mesangial Cells
MDA	Malondialdehyde
HPA	Hypothalamic‒Pituitary‒Adrenal
CRF	Corticotropin-Releasing Factor
MA	Methamphetamine
lncRNAs	Long Noncoding RNAs
ceRNA	Competitor RNA
